# Hospital Progress and Finance

**Published:** 1923-06

**Authors:** 


### HOSPITAL PROGRESS AND FINANCE.

### THE REPORT ON STREET COLLECTIONS.

THE Advisory Committee on Street Collections in the Metropolitan Police Area has issued a report from New Scotland Yard. The committee included representatives of the Charity Organisation Society, the London Mendicity Society and the London Council of Social Service. According to the report, since 1916 over a million and a-half has been collected for charities at an administrative cost of about 12½ per cent. In 1922 hospitals benefited to the extent of £109,787, a sum twice as large as that received by them in 1921. Most of the money has been collected in the streets themselves, from which it is argued that street collections do not interfere with the hospitals' ordinary sources of income. The grant of further permits is influenced by the cost of administration, so that where these are high a permit is likely to be refused. Street collections have their critics, but will it be generally admitted that they tap a new source of revenue, and do not compete with regular forms of income ?

### SEAMEN'S HOSPITALS ABROAD.

THE maintenance of the British Seamen's Hospitals abroad has come to the front with the recent discussion between representatives of the Board of Trade and the Shipping Federation in regard to the amount of the levy made on vessels arriving at Constantinople for the maintenance of the British Seamen's Hospital there. Apparently, before the war, the dues imposed were one farthing a registered ton, usually amounting to some £10 a steamer, a total said to be far in excess of the whole cost of maintenance. According to a statement in *Lloyd's List*, the hospital is to be reopened at the end of the year, and the steamship owners object to what they regard as an excessive tax, since they already pay, it seems, contributions under National Health Insurance, and also for medical attention to their crews while on articles. Subscriptions to hospitals at ports of call at home and abroad are also stated to be generous. Some shipowners propose that the expenditure should be borne by the National Health Insurance authorities ; others think this to be impracticable. The matter is of more than local interest, because there are British Seamen's Hospitals at Symrma, Port Said, Marseilles, Madeira, Rio Janeiro, Calcutta, Cape Town, and elsewhere. In these places, according to *Lloyd's List*, shipowners either subscribe annually to the hospitals or are asked to pay 2s. 6d., 5s. or 10s. on each visit of each vessel to the port. This charge, which is not compulsory, is none the less paid, and the British residents at the ports, who control these hospitals, include representatives of the shipowners. It is stated that when cases of illness among the crew are treated, a bill is usually sent by the hospital to the owner and paid by him in addition to his subscription or payment in respect of his ship's visit. The charges at Calcutta are, like those at Constantinople, said to be too high. There is, then, a *prima-facie* case for consideration. It will be a pity if each case that arises is dealt with separately, since if a general rule that can be applied appropriately to the different ports can be settled, matters will be simplified all round. A uniform principle, at least, is clearly desirable.

### THE FUTURE OF LOCAL COMMITTEES.

THE suggestions made by the Voluntary Hospitals Commission to the Devon Voluntary Hospitals Committee appear to contain the nucleus of a general policy that the commission desires these local committees to follow. Local committees are not to regard their task as limited simply to raising a sum equivalent to any grant from the balance still available. The commission says that, " While the value of local committees to the hospitals in their area can in no sense be measured by the amount of the funds which they control," the acceptance of their recommendations will obviously be facilitated if they have at their disposal a county fund. It appears that any grant from the balance at the commission's disposal is to be regarded as the nucleus of such a fund. The problem therefore is to create or increase it in each area if the local committees are to continue. The best of them have proved too useful to be allowed to fail, now that the first half of their task has been accomplished. But can they accomplish the second without injury to individual hospital appeals ?

### IN-PATIENTS AND THEIR APPROVED SOCIETIES.

The difficulty of obtaining the name of the Approved Society to which patients belong was lately brought forward at a conference between representatives of the Approved Societies and the committee of the Royal Victoria Infirmary, Newcastle. Patients conceal this information because they suppose that any grants made by the approved societies to the hospitals will interfere with the patient's sick benefit pay. The representatives of the societies then explained that it was illegal for any deduction to be made from patients because they have received treatment in a hospital. The conference has done its best to make this information public, and to remove the present erroneous impression. The Royal Victoria Infirmary has not been alone in this difficulty, and other hospitals similarly placed will be glad to know how it has been tackled.

### HOSPITAL POLICY IN SURREY.

THE Surrey and Croydon Voluntary Hospitals Committee, of which Lord Cave is chairman, and Sir A. Bingley, K.C.I.E., hon. secretary, has issued a very interesting report. The Committee asked Dr. Cates, county medical officer of health, to investigate the hospital accommodation of the area, and has accepted, in principle, his conclusions. These, briefly, are, that the number of beds in Surrey is insufficient, especially for women and children ; that facilities for treating diseases of the ear, eye, nose and throat are inadequate ; that the smaller hospitals, though valuable, suffer from piecemeal extension and unsuitable buildings; that hospital concentration is advisable for economy and efficiency both in treatment and training, and would justify an ambulance service to supply the needs of a larger area. It is interesting to note that only nine hospitals out of 32 applied to the Local Committee for a grant, because either they were not in need, or did not feel justified in asking for it. Advice was given to Mr. L. Solomon's trustees, at their request, in distributing £5,000 among Surrey hospitals, and the Committee examined, on Lord Onslow's invitation, the extension scheme of the Royal Surrey County Hospital, Guildford, and approved it and the issue of an appeal. The Committee also considered overlapping in hospital appeals, and came to the conclusion that the county hospitals would benefit if a central fund for provincial hospitals were formed on the lines of King Edward's Hospital Fund for London. The Committee is dealing with several other matters of importance, and the report, as our remarks suffice to show, is an indication of the valuable work that such local committees can perform if they make the most of their opportunities.

### DEVELOPMENTS AT SHEFFIELD.

THE Sheffield Royal Hospital, which has succeeded in paying its way for the first time for over twenty-five years, has also reduced its overdraft by £7,500. The penny-in-the-pound scheme continues to grow, but private subscriptions tend to fall. It is hoped to extend convalescent treatment, and to open two wards as soon as the extra staff can be accommodated, which the expected offer of a suitable building should make possible. An interesting plan is, when funds permit, to increase the ambulances, in order to remove not only in-patients but also to carry to and fro such out-patients as cannot walk and for whom transit by tram is not advisable. The new schemes for after-care and the transport of out-patients will be watched with interest, especially since the finances are happily improving.

### THE PLYMOUTH PROPOSAL.

HOW easy it is for objectors to find reasons for objecting is the feeling produced by the opposition of some Plymouth doctors to the penny-in-the-pound scheme that the Voluntary Hospitals Committee of the town wants to introduce. Every argument, so far as we can see, brought forward by these gentlemen has been disproved already in the successful schemes of the like nature that now abound. In the circumstances, then, the general answer is for these doctors to inform themselves of the schemes successfully established at Oxford, Sheffield, or anywhere else, and for the Voluntary Hospitals Committee of Plymouth to continue undaunted in their advocacy.

### X-RAYS AT MANCHESTER.

THE Manchester Royal Infirmary recently received a gift of £5,000 to provide an X-ray equipment, and the work has now been completed. The department consists of a demonstration room, a radioscopic room, two radiographic rooms (one of which contains a 2 k.w. high-tension Coolidge tube for dental and stereoscopic work, and the other a Potter-Bucky diaphragm), a dark room, a treatment room, and a deep therapy room. A useful feature is the workshop where repairs can be quickly executed.

### THE WAITING LIST.

SPEAKING at a Conference at York on the Care of Crippled Children, Dr. Wheatley, County Medical Officer for Shropshire, said that one of the greatest difficulties of our voluntary hospitals is that they cannot ensure that the cases most urgently needing treatment are received as speedily as possible. The system is, he said, very haphazard, and there is no means of seeing that cases are perfectly treated after they get into hospital. This seems rather harsh criticism, though all will agree with Dr. Wheatley as to the desirability of obviating the long waiting list. Dr. Wheatley insisted that hospitals should not be of an unwieldy size. Orthopædic hospitals, for instance, should not contain more than 150 beds. Since the admission of a patient is so lengthy a business, the importance of preventive measures cannot be unduly magnified, and in orthopædic work especially the health visitor and child welfare worker are absolutely essential if any real advance is to be made.

### THE CANCER QUESTION.

THE past month has given us several points of interest concerning the cancer question. First there is the welcome news that at sundry institutions where the apparatus for treatment of cancer by deeply penetrating X-rays has been installed, undoubted success has been obtained, and although the value of the Wintz rays had already been proved by the Continental workers at Erlangen, it is of first-rate importance that the apparatus is becoming available and actually used in this country. Another point, affecting the daily life of many people, comes to light in the discussion among the medical profession whether strong soaps and cosmetics tend to produce cancer. The possibility had inevitably to be borne in mind after the discovery that by constant painting of animal's skins with certain tars, extracts and so forth, cancer could be produced in them. Fortunately, the consensus of opinion seems to be that none of the materials in common daily use are in the least likely to have this dire effect upon human beings. Skin cancers are, on the whole, less common in women than in men, which is good evidence against the danger in cosmetics. As to whether cancer must, like all known infectious diseases, be introduced into a country before it can become prevalent therein, a small but interesting news item is also to hand. Pitcairn Island, a scrap of almost unvisited land of only two square miles, situated in mid-Pacific, can claim its case of cancer like every other country. It is said that a man who lived there all his days and, as far as is known, was never in contact with a cancerous person in his life, died not long ago from primary cancer of the tongue. This is yet another argument against the theory that cancer is infective.

